# Impact of Cancer-Associated Fibroblast on the Radiation-Response of Solid Xenograft Tumors

**DOI:** 10.3389/fmolb.2019.00070

**Published:** 2019-08-13

**Authors:** Alizée Steer, Nils Cordes, Verena Jendrossek, Diana Klein

**Affiliations:** ^1^Institute of Cell Biology (Cancer Research), University of Duisburg-Essen, University Hospital, Essen, Germany; ^2^Faculty of Medicine, OncoRay—National Center for Radiation Research in Oncology, Technische Universität Dresden, Dresden, Germany; ^3^Department of Radiotherapy and Radiation Oncology, Faculty of Medicine and University Hospital Carl Gustav Carus, Technische Universität Dresden, Dresden, Germany; ^4^Institute of Radiooncology—OncoRay, Helmholtz-Zentrum Dresden—Rossendorf, Dresden, Germany; ^5^German Cancer Consortium (DKTK), Partner Site Dresden, Heidelberg, Germany; ^6^German Cancer Research Center (DKFZ)—Partner Site Dresden, Heidelberg, Germany

**Keywords:** cancer-associated fibroblast, fibroblast, cancer therapy, radiation therapy, radiation resistance, tumor stoma, tumor microenvironment

## Abstract

Increasing evidence indicates that the heterogeneous tumor stroma supports therapy resistance at multiple levels. Fibroblasts, particularly cancer-associated fibroblasts (CAFs) are critical components of the tumor stroma. However, the impact of CAFs on the outcome of radiotherapy (RT) is poorly understood. Here, we investigated if and how fibroblasts/CAFs modulate the radiation response of malignant tumors by altering cancer cell radiosensitivity or radioresistance *in vitro* and *in vivo*. The influence of fibroblasts on cancer cell proliferation, cell death induction and long-term survival after RT was studied using different sets of fibroblasts and cancer cells in an indirect co-culture (2D) system to analyse potential paracrine interactions or a 3D model to study direct interactions. Paracrine signals from embryonic NIH-3T3 fibroblasts promoted MPR31.4 prostate and Py8119 breast cancer cell proliferation. Indirect co-culture with L929 skin fibroblasts induced higher levels of apoptosis in irradiated MPR31.4 cells, while they promoted proliferation of irradiated Py8119 cells. In addition, NIH-3T3 fibroblasts promoted long-term clonogenic survival of both tumor cell types upon irradiation in the 3D co-culture system when compared to non-irradiated controls. Also *in vivo*, co-implantation of cancer cells and fibroblasts resulted in different effects depending on the respective cell combinations used: co-implantation of MPR31.4 cells with NIH-3T3 fibroblasts or of Py8119 cells with L929 fibroblasts led to increased tumor growth and reduced radiation-induced tumor growth delay when compared to the respective tumors without co-implanted fibroblasts. Taken together, the impact of fibroblasts on cancer cell behavior and radiation sensitivity largely depended on the respective cell types used as they either exerted a pro-tumorigenic and radioresistance-promoting effect, an anti-tumorigenic effect, or no effect. We conclude that the plasticity of fibroblasts allows for such a broad spectrum of activities by the same fibroblast and that this plasticity is at least in part mediated by cancer cell-induced fibroblast activation toward CAFs.

## Introduction

Genetic and cell-biology studies indicate that tumor growth is not just determined by cancer cells themselves, but it is also suppoted by the tumor's stromal microenvironment (Pietras and Östman, 2010). Apart from cancer cells, the tumor stroma comprises fibroblasts, cells of the immune system, vascular endothelial cells, pericytes and smooth muscle cells, mesenchymal cells and adipocytes, as well as the extracellular matrix. Among the stromal cells, the tumor-associated fibroblasts, also termed cancer-associated fibroblasts (CAFs), have been identified as one of the most active cell type (Kalluri and Zeisberg, 2006; Franco et al., 2010; Leef and Thomas, 2013). These cells are found in almost all solid cancers, but their relative abundance differs. Breast, prostate, and pancreatic cancers contain high numbers of CAFs, whereas brain, renal, and ovarian cancers bear a lower fibroblastic content (Neesse et al., 2011; Smith et al., 2013). Under physiological conditions, the stroma is an important barrier to the malignant transformation of cells. However, during neoplastic transformation the cellular components of the tumor stroma become activated enlivening cancer cell invasiveness, progression, and therapy resistance (Hanahan and Weinberg, 2000; Hanahan and Robert Weinberg, 2011). As the tumor evolves, continuous tumor-stroma-cell communication synergistically supports and augments tumor growth by creating a dynamic signaling exchange (Bissell and Radisky, 2001; Furuta et al., 2011). Herein, transforming growth factor beta (TGF-β) and interleukin (IL)-1 beta signaling have been shown to transform fibroblasts into activated myofibroblasts/ CAFs (Lewis et al., 2004; Dudás et al., 2011). Cytokines including platelet-derived growth factor (PDGF), IL-4, IL-6 or prostaglandin E (PGE) have also been reported to induce fibroblast-to-CAF differentiation (Hawinkels et al., 2014; Kaler et al., 2014). CAFs in turn modulate the response of solid tumors to cancer therapy (Duluc et al., 2015; Zhang et al., 2015; Hesler et al., 2016). The ability of cancer cells to evade programmed cell death is at least in part derived from survival signals supplied by CAF (Kalluri, 2016).

The majority of cancer patients receive combinations of treatment including surgery, radiotherapy, chemotherapy, immunotherapy, and/or targeted therapy with the ultimate goal to achieve optimal tumor control and enhance survival of cancer patients with limited impact on their quality of life (Ito et al., 2011). Herein, radiotherapy (RT) is an essential part of effective standard cancer treatment protocols: 50 to 60% of patients receive radiotherapy in their treatment schedules although radio-resistance still occurs. Over the past two decades, the radiobiologists view changed and recognized that the tumor microenvironment and particularly the stromal cell compartment are of central importance for the radiotherapy response and therapy outcome (Durand, 1994; Barcellos-Hoff et al., 2005; Demaria et al., 2005). Own previous work revealed that e.g., radiation resistance of prostate cancer cells can be linked to the loss of the membrane protein caveolin-1 in stromal fibroblasts (Klein et al., 2015; Panic et al., 2017; Ketteler and Klein, 2018). These observations suggest that some factors derived from stromal fibroblasts may be involved in mediating radiation resistance, while being supposed tumor-suppressive in the healthy situation. In addition, RT itself might promote activation of fibroblasts in normal tissues and/or at the tumor margins toward a CAF-like phenotype and thus modulate the behavior of resident fibroblasts/CAFs for the response to RT. However, the role of CAFs in the response to radiotherapy is still largely unknown. The identification of processes and involved pathways that drive stroma-mediated resistance and more specifically the effects mediated by CAFs at advanced tumor stages will help to develop of novel and effective strategies to target therapy resistance and improve the treatment outcome (Bonomi et al., 2015; Dauer et al., 2018). For example, inactivation of CAFs via the inhibition of the TGF-β signaling pathway was shown to promote regression of pancreatic cancer (Dauer et al., 2018). The ability to target FAP in the stroma of advanced or metastatic FAP-positive cancer with repeated infusions of a humanized antibody (sibrotuzumab) directed against FAP provided additional evidence to inhibit CAF functions in the targeting and therapy of epithelial malignancies (Scott et al., 2003; Jiang et al., 2016). In contrast, CAFs depletion accelerated tumor growth and reduced survival in pancreatic cancer pointing to tumor-suppressive effects of CAFs (Ozdemir et al., 2014).

Thus, the role of CAFs in tumor growth and progression is still controversial and their role in the radiotherapy response remains to be explored. The central aim of this work was to investigate how CAFs modulate the radiation response of tumors among others by altering cancer cell proliferation, survival and radiation response. We further investigated whether ionizing radiation itself might enhance the growth- and resistance-promoting properties of fibroblasts and respective CAFs. Therefore, we used a systematic approach to determine and specify how fibroblasts modulate the radiation response of tumor cells and if this modulation can be linked to the fibroblasts/CAFs phenotype.

## Materials and Methods

### Cells

The mouse prostate epithelial cell line MPR31.4 was a kind gift of TC Thompson, Scott (Department of Urology, Baylor College of Medicine, Houston, USA) (Thompson et al., 1993; Shaker et al., 2000). The mouse breast cancer cell line Py8119, the skin and embryonic fibroblasts cell lines L929, NIH-3T3 were purchased from ATCC (Manassas, VA, USA).

### Mouse Tumor Model

C57BL/6 wild-type (WT) mice were bred and housed under specific-pathogen-free conditions in the animal facilities laboratory of the University Hospital Essen. Food and drinking water were provided *ad libitum*. All protocols were approved by the universities' animal protection boards in conjunction with the legal authority (LANUV Düsseldorf) according to German animal welfare regulations and by the Committee on the Ethics of Animal Experiments of the responsible authorities [Landesamt für Natur, Umwelt und Verbraucherschutz (LANUV), Regierungspräsidium Düsseldorf Az.84-02.04.2014.A244; Az.84-02.04.2015.A586; Az.81-02.04.2018.A158; Az. 81-02.04.2018.A267]. Mouse xenograft tumors were generated by subcutaneous injection of 0.25 × 10^6^ or 0.125 × 10^6^ cancer cells (MPR31.4 or Py8119) either alone or mixed with 0.25 × 10^6^ or 0.125 × 10^6^ fibroblasts cells (NIH-3T3 or L929) into the hind limb of mice (total volume 50 μl) as previously described (Panic et al., 2017). Up to 20 animals of each experimental group received a single subcutaneous injection of 0.5 × 10^6^ or 0.25 × 10^6^ viable cells.

### Tumor Irradiations

Mice were anesthetized with 2% isoflurane and irradiated in 0.8% isoflurane with either a single dose of 0 Gy (sham control treatment) or with 10 Gy ±5% in 5 mm tissue depth (~1.53 Gy/min, 300 kV, filter: 0.5 mm Cu, 10 mA, focus distance: 60 cm) using a collimated beam with a XStrahl RS 320 cabinet irradiator (XStrahl Limited, Camberly, Surrey, Great Britain) (Panic et al., 2017). Mice were humanely sacrificed at indicated time points with CO_2_ inhalation and transcardial perfusion, and tumor tissue was isolated for respective downstream analysis.

### Preparation of Tumor for Paraffin Sections

Tumors were fixed overnight at 4°C in 4% PFA in PBS, pH 7.2 and placed in embedding cassettes. After, dehydration in 70% ethanol, PFA-fixed tumors were processed using automated standard procedures and subsequently embedded in paraffin. Five micrometer tissues sections obtained with Leica microtome were mounted on coated microscope slides.

### Immunohistochemistry and Histology

Paraffin-embedded tissue sections were hydrated using a descending alcohol series, incubated for 10–20 min in target retrieval solution (Dako, Glostrup, Denmark) and incubated with blocking solution (2% fetal calf serum/phosphate-buffered saline). After permeabilization, sections were incubated with primary antibodies over night at 4°C. Antigen was detected with a peroxidase-conjugated secondary antibody (1/250) and DAB staining (Dako). Nuclei were counterstained using hematoxylin. Tumor sections were stained with hematoxylin and eosin and Masson Goldner Trichrome (Carl Roth Karlsruhe) as previously described (Klein et al., 2017; Panic et al., 2017).

### Indirect (Transwell) Co-culture

Cancer cells were plated in 6 wells plates ensuring that after 72 h the cells were not over-confluent. Transwells were added on the top of the cancer cells and fibroblasts were plated into each transwell at the same cell concentration (ratio 1+1) or two times more (ratio 1+2).

### Irradiation of Cell Cultures

Radiation with indicated doses was performed using the Isovolt-320-X-ray machine (Seifert–Pantak, East Haven, CT) at 320 kV, 10 mA with a 1.65-mm aluminum filter, and a distance of about 500 mm to the object being irradiated (Klein et al., 2015). The effective photon energy was about 90 kV, and the dose rate about 3 Gy/min.

### Cell Viability Assay

The number of living cells was determined upon staining of the cells with the vital dye trypan blue. For this, cells were harvested with Trypsin-EDTA, re-suspended in fresh medium, diluted with trypan blue, and counted employing a Neubauer chamber.

### Flow Cytometry Analysis

For quantification of apoptotic DNA-fragmentation (sub-G1 population), cells were incubated for 15–30 min with a staining solution containing 0.1% (w/v) sodium citrate, 50 μg/ml PI, and 0.05% (v/v) Triton X-100 (v/v) and subsequently analyzed by flow cytometry (FACS Calibur, Becton Dickinson, Heidelberg, Germany; FL-2) (Klein et al., 2015; Panic et al., 2017).

### 2D Colony Formation Assay

Clonogenic cell survival was tested in response to ionizing radiation with radiation doses between 0 Gy (control) and 10 Gy. Cancer cells were seeded in 6-well plates and fibroblasts in transwell chambers. Cells were irradiated 24 h after seeding (5, 7.5, 10 Gy) and further incubated under standard culturing conditions. Plates were incubated for a total of 7 days to allow growth of single colonies. For determination of colony formation cells were fixed in 3.7% formaldehyde and 70% ethanol, stained with 0.05% Coomassie Brillant blue. Colonies of at least 50 cells were counted.

### Generation of Labeled Cells

Transfection mixtures containing plasmid pEGFP-N1 (Addgene) or pTagRFP-N (Evrogen) and lipofectamine in OptiMEM were incubated 1 h at 37°C on MPR31.4, Py8119 and NIH-3T3 cells. Then, the cells were sorted by flow cytometry and selected with G418 antibiotics.

### 3D Colony Formation Assay

Measurement of 3D cell survival was accomplished as reported before. In brief, single cancer cells alone or with fibroblasts were co-plated into a mixture of 1 mg/ml high concentration extracellular matrix (Corning® Matrigel® Basement Membrane Matrix High Concentration (HC), ^*^LDEV-free, Product Number 354248) in 96-well plates. The cells were irradiated (3, 6, 9 Gy) 24 h after seeding and colonies (>50 cells) were microscopically counted 7 days after IR (2.5 magnification). In addition, colonies picture were taken by fluorescence microscopy (RFP, GFP) at 10-fold magnifications to determine the composition of the colonies (Eke et al., 2015).

### Real-Time Reverse Transcription PCR (qRT-PCR)

RNA was isolated using RNeasy Mini Kit (74106, Qiagen, Hilden, Germany) according to the manufacturer's instruction and as previously described (Panic et al., 2017; Ketteler et al., 2019). Expression levels were normalized to the reference gene (β-actin; set as 1) and were shown as relative quantification. Specific primers were designed using Primer 3 (http://bioinfo.ut.ee/primer3-0.4.0/) based on available NCBI nucleotide CDS sequences and all primers used were intron-spanning. qRT-PCR was carried out using specific oligonucleotide primers (s sense, as antisense; list primers like pdgfrβs CCTGTGCAGTTGCCTTACGA, pdgfrβas TCTCGCTACTTCTGGCTGTC, ng2/Cspg4s GCTGTGCGTCGTTTGAGTTT, ng2/Cspg4as CAACAAACAGCCCATCTGCC, α-sma/Acta2s ACGGCCGCCTCCTCTTCCTC, α-sma/Acta2as GCCCAGCTTCGTCGTATTCC, tgfβ1s GAACCAAGGAGACGGAATACAG, tgfβ1as AACCCAGGTCCTTCCTAAAGTC, snai-1s TCAACTGCAAATATTGTAACAAGGA, snai-1as CTGGCACTGGTATCTCTTCACA, snai-2s TCCTTCCTGGTCAAGAAACATT, snai-2as TGTGATCCTTGGATGAAGTGTC, β-actins CCAGAGCAAGAGAGGTATCC and β-actinas CTGTGGTGGTGAAGCTGTAG as previously described (Panic et al., 2017).

### Statistical Analysis

If not otherwise indicated, data were obtained from 3 independent experiments with at least 2–3 mice each. Total mice numbers were stated in the figure legends. Statistical significance was evaluated by 1- or 2-way ANOVA followed by Tukey's or Bonferroni multiple comparisons *post-hoc* test and set at the level of *P* ≤ 0.05. Data analysis was performed with Prism 5.0 software (GraphPad, La Jolla, California).

## Results

### Fibroblasts Differentially Affect Cancer Cell Proliferation, Short-Term, and Long-Term Survival in Indirect Co-culture

**Figure 1:**
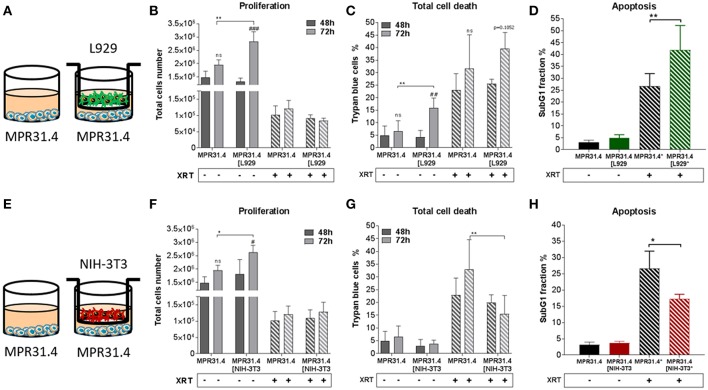
NIH-3T3 embryonic fibroblasts increased proliferation and reduced radiation-induced cell death of MPR31.4 prostate cancer cells when L929 skin fibroblasts increased radiation-induced MPR31.4 cell death. MPR31.4 cancer cells were cultured alone or together with stromal fibroblasts (in indirect co-culture) for 24 h prior to irradiation with 0 or 10 Gy (ratio 1+1, **A–E**). After 48 and 72 h, total cell numbers as well as dead cells were counted by trypan blue **(B–F, C–G)**. ^*^p < 0.05, ^**^p < 0.01, ^***^p < 0.005, ^****^p < 0.001 analyzed by two-way ANOVA test followed by Tukey's test, compared cancer cells with fibroblasts to cancer cells cultured alone from three independent experiments (means ± SD). “ns” present for no significant, ^#^*p* < 0.05, ^##^*p* < 0.01, and ^###^*p* < 0.5 analyzed by two-way ANOVA test followed by Tukey's test, compared 72-48 h. SubG1 fractions were measured by Nicoletti staining, 72 h after irradiation **(D–H)**. ^*^p < 0.05 and ^**^p < 0.01 analyzed by one-way ANOVA test followed by Tukey's test from three independent experiments (means ± SD).

**Figure 2:**
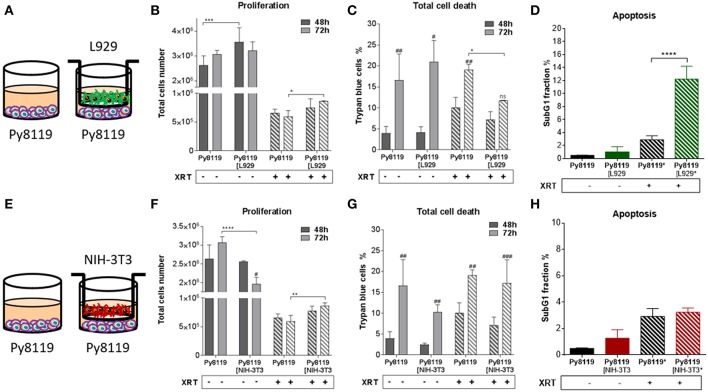
Fibroblasts increased proliferation of Py8119 breast cancer cells after radiation when L929 skin fibroblasts reduced radiation-induced Py8119 cell death, NIH-3T3 embryonic fibroblasts had no impact on it. Py8119 cancer cells were cultured alone or together with stromal fibroblasts (in indirect co-culture) for 24 h prior to irradiation with 0 or 10 Gy (ratio 1+1, **A–E**). After 48 and 72 h, total cell numbers as well as dead cells were counted by trypan blue **(B–F, C–G)**. ^*^p < 0.05, ^**^p < 0.01, and ^***^p < 0.001 analyzed by two-way ANOVA test followed by Tukey's test, compared cancer cells with fibroblasts to cancer cells cultured alone from three independent experiments (means ± SD). “ns” present for no significant, ^#^*p* < 0.05, ^##^*p* < 0.01, ^###^*p* < 0.005 analyzed by two-way ANOVA test followed by Tukey's test, compared 72-48 h. SubG1 fractions were measured by Nicoletti staining, 72 h after irradiation **(D–H)**. ^****^p < 0.0001 analyzed by one-way ANOVA test followed by Tukey's test from three independent experiments (means ± SD).

**Figure 3:**
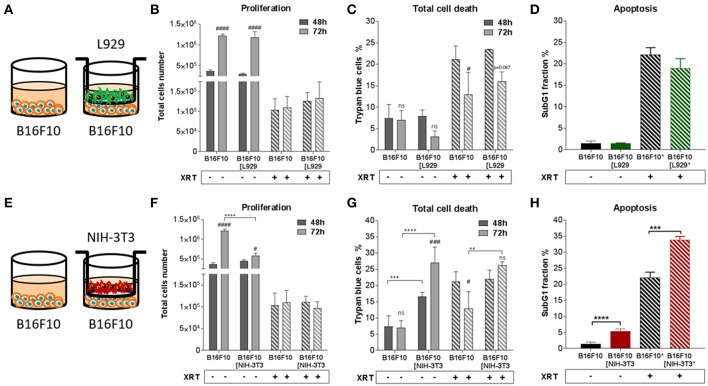
Fibroblasts didn't proliferation of B16F10 melanoma cancer cells after radiation when NIH-3T3 embryonic fibroblasts increased radiation-induced B16F10 cell death, L929 skin fibroblasts had no impact on it. B16F10 cancer cells were cultured alone or together with stromal fibroblasts (in indirect co-culture) for 24 h prior to irradiation with 0 or 10 Gy (ratio 1+1, **A–E**). After 48 and 72 h, total cell numbers as well as dead cells were counted by trypan blue **(B–F, C–G)**. ^**^p < 0.01, ^***^p < 0.005, ^***^p < 0.001 analyzed by two-way ANOVA test followed by Tukey's test, compared cancer cells with fibroblasts to cancer cells cultured alone from three independent experiments (means ± SD). “ns” present for no significant, ^#^*p* < 0.05, ^###^*p* < 0.005, ^####^*p* < 0.001 analyzed by two-way ANOVA test followed by Tukey's test, compared 72-48 h. SubG1 fractions were measured by Nicoletti staining, 72 h after irradiation **(D–H)**. ^***^p < 0.005 and ^****^p < 0.001 analyzed by one-way ANOVA test followed by Tukey's test from three independent experiments (means ± SD).

**Figure 4:**
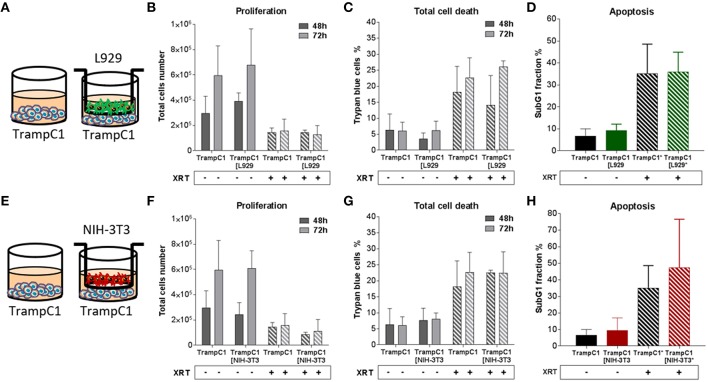
Fibroblasts didn't influence TrampC1 prostate cancer cells proliferation and radiation-induced cell death. TrampC1 cancer cells were cultured alone or together with stromal fibroblasts (in indirect co-culture) for 24 h prior to irradiation with 0 or 10Gy (ratio 1+1, **A–E**). After 48 and 72 h, total cell numbers as well as dead cells were counted by trypan blue **(B–F, C–G)**. SubG1 fractions were measured by Nicoletti staining, 72 h after irradiation **(D–H)**.

In order to investigate how fibroblasts and respective CAFs modulate the radiation response of cancer cells, different types of fibroblasts were first investigated *in vitro* in an indirect co-culture with different cancer cells (Figures 1–**4**). Cancer cells from diverse tissue origins were selected with regard to the knowledge that breast and prostate tumors are known to contain a high number of CAFs. Therefore, the respective cancer cells (MPR31.4, Py8119, B16F10 and TrampC1) were co-cultured indirectly with fibroblasts (L929 and NIH-3T3) using transwell chamber culture dishes (ratio 1+1) allowing only the exchange of soluble factors. Cancer cell proliferation, total cell numbers and apoptosis were determined 72 h after treatment with 0 or 10 Gy. The data of these investigations are depicted in Figures 1–**4**.

Differential effects of the same fibroblast on different cancer cells were observed (summarized in Table 1). Some of the cancer cells investigated were sensitive to proliferation-promoting effects of soluble factors of specific fibroblasts. These include MPR31.4 (L929; NIH-3T3; Figures 1B,F) and Py8119 (L929; Figure 2B). In contrast the following cells revealed reduced proliferation in the presence of indirect co-culture with fibroblasts: Py8119 (NIH-3T3; Figure 2F) and B16F10 (NIH-3T3; Figure 3F). Surprisingly, untreated MPR31.4 and B16F10 cells showed increased levels of total cell death when indirectly co-cultured with L929 cells or NIH3T3 fibroblasts, respectively (Figures 1C, 2C,G, 3G; Table 1) suggesting sensitivity to cell death induced by factors released from the fibroblasts. In MPR31.4 cells (L929; Figure 1D) and B16F10 cells (NIH-3T3; Figure 3H) this was associated with increased apoptosis levels (Table 1). Furthermore, heterogeneity in the influence of soluble factors from fibroblasts on the irradiated cancer cells proliferation and survival were also observed. After radiation, indirect co-culture with L929 cells increased total cell death (by trend) and apoptosis of MPR31.4 cells (Figures 1C,D) as well as indirect co-culture of NIH-3T3 cells increased total cell death and apoptosis of B16F10 cells (Figures 3G,H). However, indirect co-culture of NIH-3T3 cells with MPR31.4 and L929 cells with Py8119 had opposite effects (Figures 1G,H, 2C,D). Of note, no impact of the fibroblasts was observed for TrampC1 ells (Figure 4).

**Table 1:** Multiple effects of fibroblasts on the radiation response of tumor cells.

**Cancer cells**	**Fibroblasts**	**Indirect co-culture**					**Effect**
		**Short-term**				**Long-term**	
		**Proliferation**		**Cell death**		**CFA**	
		**0 Gy**	**10 Gy**	**0 Gy**	**10 Gy**		
MPR31.4	NIH-3T3	↗	–	–	↘	(trend)↗	Tumor promoting
MPR31.4	L929	↗	–	↗	↗	–	Tumor suppressing
Py8119	NIH-3T3	↘	↗	–	–	↗	Controversial /No effect
Py8119	L929	↗	↗	–	↘	↗	Tumor promoting
B16F10	NIH-3T3	↘	–	↗	↗	-	Tumor suppressing
B16F10	L929	–	–	–	–	↗	No effect
TrampC1	NIH-3T3	–	–	–	–	n.d	No effect
TrampC1	L929	–	–	–	–	n.d	No effect

*NIH-3T3 fibroblasts induced a radio-sensitizing effect on B16F10 melanoma cells, while promoting radio-resistance on MPR31.4 prostate carcinoma cells. L929 fibroblasts induced on MPR31.4 cells a radio-sensitizing effect, while promoting radio-resistance of Py8119 breast cancer cells. Minus (-) stands for “no effect”. n.d. not determined*.

Thus, the presented results strongly suggest that the impact of fibroblasts on the tumor cell radiation response largely depends on the fibroblast-tumor cell combination. Fibroblast exerted either tumor-suppressing, tumor-promoting, radiation response-modulating effects or no effect.

**Figure 5:**
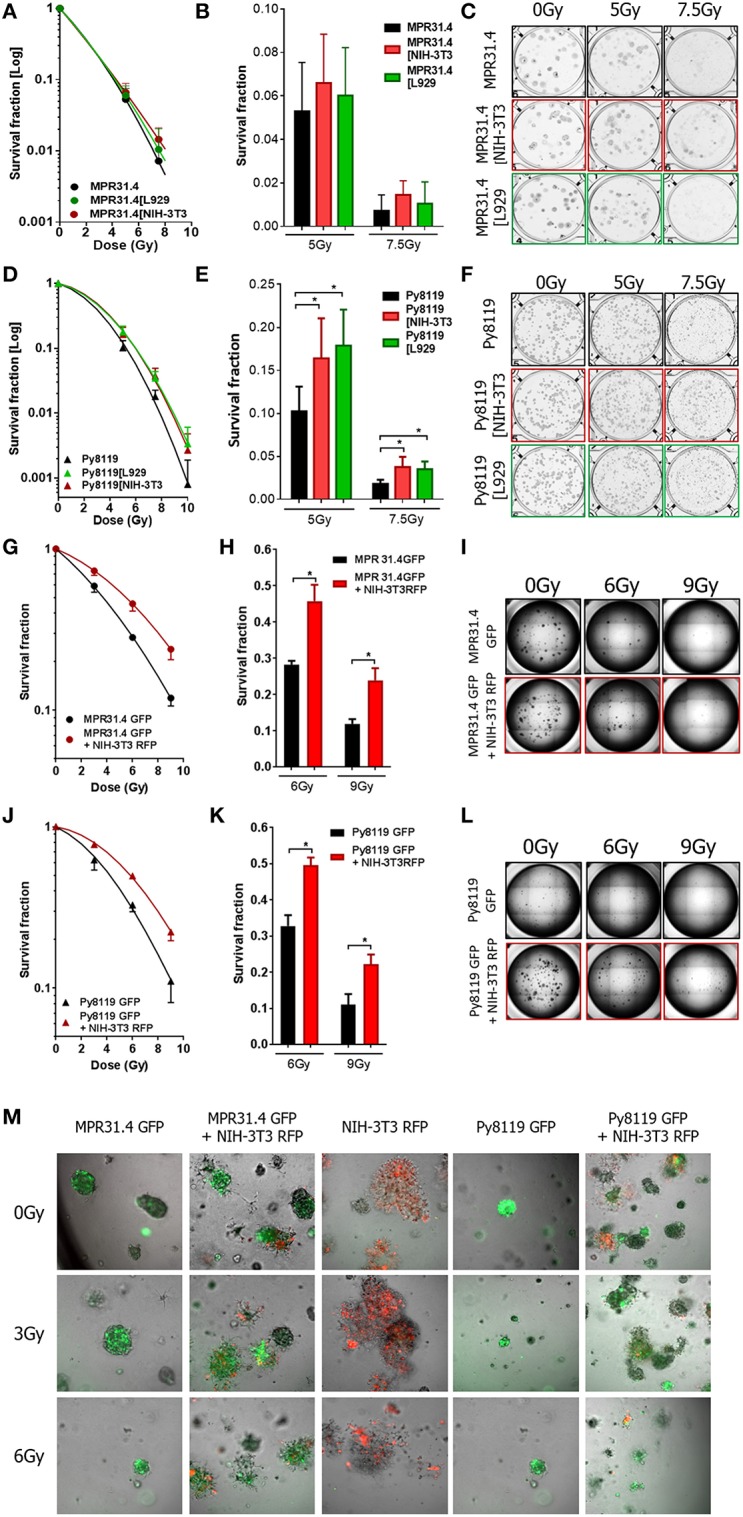
Fibroblasts increased differentially the radiation long term survival of prostate and breast cancer cells in indirect and 3D direct co-culture. MPR31.4 or Py8119 cancer cells were plated alone or in co-culture (transwell) with stromal fibroblasts for colony formation assay, irradiated with indicated doses (0–10 Gy) and subsequently further incubated for additional 7 days **(A–F)**. Graphs depict the surviving fractions from one representative experiment measured in sextuplet of two independent experiments (means ± SD) **(A,B,D,E)**. The plates were scanned and colonies were counted **(C,F)**. ^*^p < 0.5 by one-way ANOVA test followed by Tukey's test. MPR31.4 or Py8119 cancer cells were plated for colony formation assay alone or with NIH-3T3 fibroblasts [ratio (1+1)] in a 3D Matrigel system, irradiated with indicated doses (0–9 Gy) and subsequently further incubated for additional 7 days **(G–M)**. Surviving fractions from three experiments measured in quintuplet each were shown (means ± SEM) **(G,J)** the colonies were counted and the survival fraction calculated **(H,K)**. Picture of the well-taken in bright light at 2.5 magnifications **(I,L)**. Representative pictures of the well taken by fluorescence microscopy at 10 magnifications **(M)**. ^*^p < 0.5, by two-way ANOVA test followed by Tukey's test.

Next, the indirect co-culture system was used to study the relevance of the above findings for radiosensitivity and determined by long-term survival of MPR31.4, Py8119, and B16F10 cells using standard 2D colony formation assay (CFA; 0 to 10 Gy). In contrast to the results from the short-term investigations co-culture with L929 fibroblasts or NIH-3T3 fibroblasts did not significantly alter long-term survival of MPR31.4 (Figures 5A,C) or B16F10 cells, respectively, (Supplementary Figures 1A–D). Moreover, despite differential effects on short-term survival of irradiated Py8119 cells the presence of NIH-3T3 or L929 fibroblasts significantly enhanced the long-term survival of Py8119 cancer cells (Figure 5D–F). This is exemplarily shown in detail for the survival fractions at 7.5Gy (Figure 5E). No significant differences were observed in the cells plating efficiencies (data not shown).

### Fibroblasts Alter the Long-Term Survival of Prostate and Breast Cancer Cells in 3D Direct Co-culture

Earlier work revealed that fibroblasts/CAFs communicate with cancer cells by paracrine signal via small molecules like cytokines but also by direct cell-to-cell interaction (Alkasalias et al., 2018). To extend the effects observed in indirect co-culture, the direct interactions between stromal fibroblasts and cancer cells were investigated in addition using a 3D co-culture system. Specifically, we wanted to confirm the resistance-promoting action of NIH-3T3 (in combination with MPR31.4 cells) as well as L929 fibroblasts (with Py8119). To allow discrimination of cancer cells and fibroblasts in these co-cultures MPR31.4 and Py8119 cancer cells were transfected with a pEGF-N1 vector encoding GFP, whereas NIH-3T3 fibroblasts were labeled with a pTagRFP-N vector encoding RFP. Flow cytometry-sorted fluorescence-positive and antibiotics selected cells were then used.

MPR31.4 and Py8119 GFP-tagged cells were then cultured alone or in presence of NIH-3T3-RFP fibroblasts (ratio 1+1) in a self-established 3D Matrigel co-culture system (Figures 5G–M). Colonies were observed by bright field and fluorescence microscopy 7 days after XRT (Figures 5I,L,M). Formed colonies were quantified and cancer cell clonogenic survival was calculated (Figures 5G,H,J,K). Co-culture with NIH-3T3 fibroblasts enhanced the clonogenic survival of MPR31.4 and Py8119 cells upon radiation as compared to cancer cells alone. Cell plating efficiency was calculating for all conditions and revealed that NIH-3T3 cells enhanced the MPR31.4-related formation of colonies as compared to MPR31.4 cultured alone (Supplementary Figure 1E). When co-cultured with NIH-3T3 fibroblasts, the survival fraction of Py8119 cancer cells increased, which indicates an increase of radiation resistance of Py8119 cancer cells. Of note, NIH-3T3 did not influence Py8119 formation of colonies compared to Py8119 cultured alone (Supplementary Figure 1F). To exclude colonies composed only of fibroblasts in the co-cultures, the composition of the colonies was evaluated by fluorescence microscopy (Figure 5M). In the direct co-culture conditions, colonies were composed either of a mixture of cancer cells and fibroblasts (GFP and RFP signal) or only of cancer cells (GFP). Colonies composed only of fibroblasts (RFP) were not found. Of note, fibroblasts cultured alone (Figure 5M, middle column) did not form regular colonies.

As L929 fibroblasts strongly clustered upon 3D culturing (even when using reduced cell numbers), the effect of L929 on cancer cells colony formation in direct co-culture could not be investigated. In summary, the direct interaction of NIH-3T3 fibroblasts with MPR31.4 prostate or Py8119 breast cancer cells promoted the long-term survival of the irradiated cancer cells in 3D co-culture *in vitro*. Conclusively, the resistance-promoting action of NIH-3T3 fibroblasts observed upon indirect co-culture was confirmed upon direct 3D co-culture. In contrast to the observed findings for the quite resistant Py8119 cells upon indirect-co-culturing with NIH-3T3 fibroblasts (no effect), we observed a resistance-promoting action of NIH-3T3 fibroblasts upon direct 3D co-culture.

### Induction of CAF Marker Proteins in Fibroblasts by Cancer Cells and Ionizing Radiation

**Figure 6:**
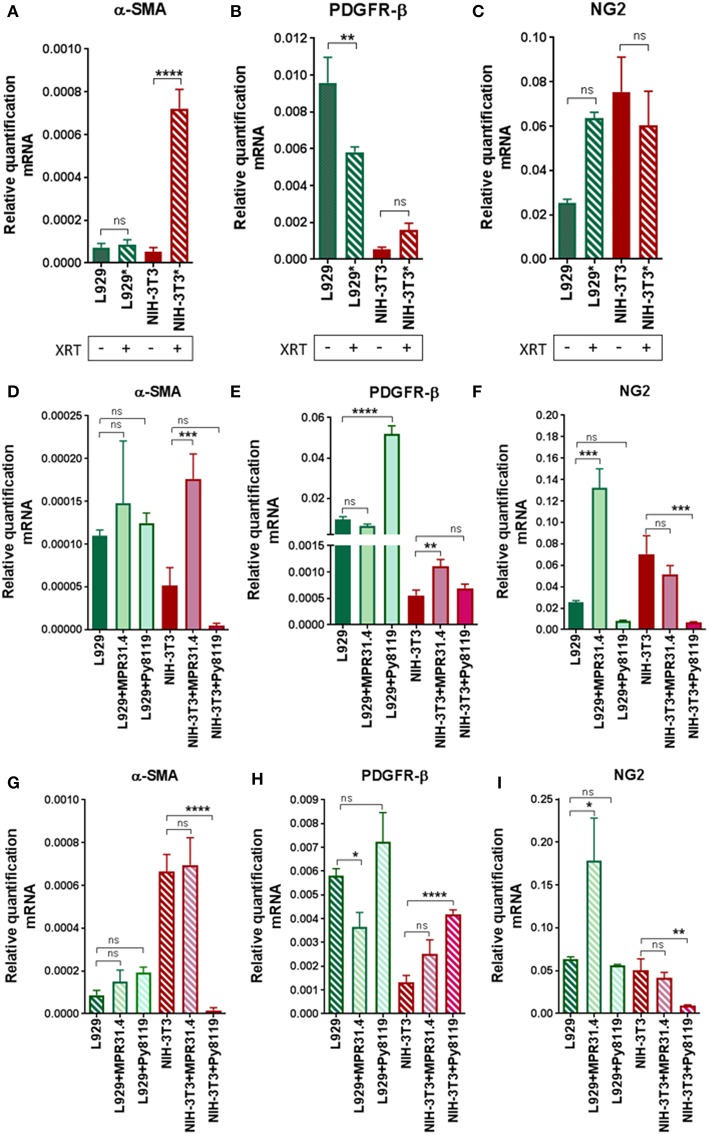
Fibroblasts did not express a CAFs-like phenotype 72 h after radiation in indirect co-culture with cancer cells. L929 or NIH-3T3 stromal fibroblasts alone **(A–C)** or together with MPR31.4 or Py8119 cancer cells [transwell, (ratio 1+1)] were cultured 24 h prior irradiation with 0 **(D–F)** or 10Gy **(G–I)**. After 72 h, qRT-PCR analysis of the CAFs markers α-SMA (Acta2), PDGFR-β and NG2 were performed in total RNA isolates of cultured fibroblasts. Respective expression levels were normalized to β-actin (set at 1). Shown are mean values ± SEM from 3 independent samples per group measured each in triplicate each. “ns” present for no significant and ^*^p < 0.05, ^**^p < 0.01, ^***^p < 0.001, ^****^p < 0.0001 analyzed by one-way ANOVA test followed by Tukey's test.

In order to investigate if radiation itself and/or respective cancer cells were able to activate fibroblasts into a pro-tumorigenic CAF phenotype, we quantified typical CAFs markers (α-SMA, PDGFR-β, and NG2) in mRNA isolates of NIH-3T3 and L929 fibroblasts after the indirect co-culture with cancer cells with or without radiation treatment using quantitative Real Time RT-PCR (qRT-PCR) (Figure 6). Radiation alone was not sufficient to induce a CAF phenotype in L929 and NIH-3T3 fibroblasts (Figures 6A–C). Radiation induced a decrease of PDGFR-β of approximately 1.5-fold and no change of α-SMA and NG2 in L929 fibroblasts. Instead, radiation induced an increase of α-SMA expression by about 7-fold in NIH-3T3 fibroblasts when compared to non-irradiated controls, whereas NG2 and PDGFR-β expression was not significantly changed. Thus, radiation alone induced a variable change in the fibroblast phenotype of *in vitro* cultured fibroblasts, at least in the indicated time frame.

Next, the effect of the cancer cells on the fibroblast's phenotype was analyzed (Figures 6D–F). MPR31.4 induced a decrease in PDGFR-β expression by about 1.5-fold, an increase in NG2 of about 5-fold and did not affect the expression of α-SMA in L929 when compared to L929 cultured alone. In contrast to our expectations, Py8119 did not influence α-SMA and NG2 levels in L929 despite PDGFR-β expression was increased by about 5-fold. Moreover, MPR31.4 did not influence PDGFR-β and NG2 levels in NIH-3T3 although it increased α-SMA expression levels by about 3.5-fold in NIH-3T3 cells. Furthermore, Py8119 induced a decrease in α-SMA expression by about 10-fold and in NG2 expression by about 7-fold and did not change the expression of PDGFR-β in NIH-3T3. Therefore, cancer cells were able to affect the fibroblast phenotype in indirect co-culture.

Finally, the effect of the cancer cells on the fibroblasts' phenotype after radiation was determined (Figures 6G–I). MPR31.4 did not influence α-SMA, PDGFR-β and NG2 levels in NIH-3T3 fibroblasts after IR. In L929 fibroblasts, after IR, MPR31.4 induced an increase of NG2 about 3.5-fold, a decrease of PDGFR-β (~1.5-fold) and did not influence α-SMA levels. Furthermore, Py8119 induced a significant decrease in α-SMA expression and in NG2 expression and increase the expression of PDGFR-β in NIH-3T3. In L929, Py8119 did not significantly influence α-SMA, PDGFR-β and NG2 levels after IR.

In conclusion, fibroblasts co-cultured indirectly with cancer cells did not undergo a significant up-regulation of known CAFs markers. This might indicate that an indirect co-culture with cancer cells is not sufficient to induce a CAF-like phenotype.

### NIH-3T3 Fibroblasts Induce Radiation Resistance of MPR31.4 Prostate Xenograft Tumors, While L929 Fibroblasts Fosters Radiation Resistance of Py8119 Breast Xenograft Tumors

**Figure 7:**
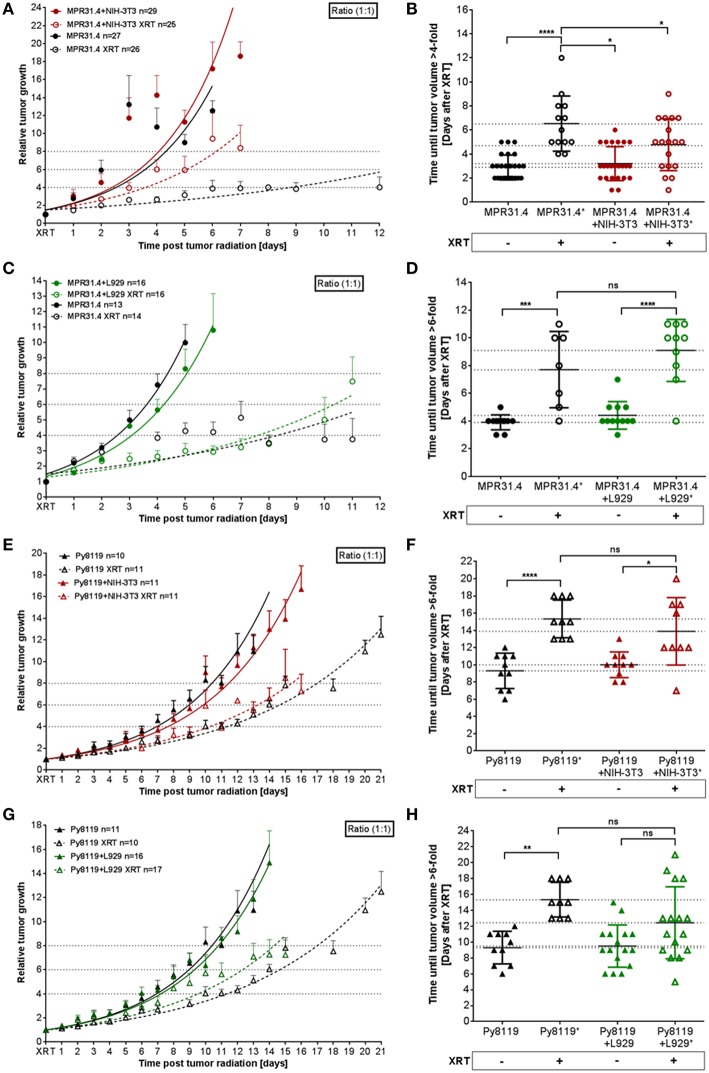
Stromal L929 fibroblasts and NIH-3T3 fibroblasts, respectively induced radio-resistance of breast and prostate tumors. MPR31.4 prostate **(A–D)** and Py8119 breast **(E–H)** cancer cells alone or together with NIH-3T3 embryonic **(A,B,E,F)** L929 skin **(C,D,G,H)**, or fibroblasts [ratio of (1+1)] were subcutaneously co-implanted onto the hint leg of C57BL/6 mice. When tumor volumes of 100 mm3 were reached, one group received a single radiation dose of 10 Gy to the tumor. The tumor volume was determined at indicated time points (left diagram). Data were represented as mean ± SEM from 2 to 3 independent experiments (in total 10-27 mice). Tumor growth and respective tumor growth delay were determined as time (days) until the 4–6-fold volume was reached (right diagram). ^*^p < 0.05, ^***^p < 0.001, ^****^p < 0.0001 by one-way ANOVA followed by Tukey's test.

To investigate a potential relevance of the *in vitro* findings *in vivo*, MPR31.4 prostate or Py8119 breast cancer cells were implanted subcutaneously either alone or in combination with stromal L929 or NIH-3T3 fibroblasts (ratio 1+1) onto immune competent C57BL/6 mice; both combinations which were shown to result in increased radio-resistance. Tumor growth and growth retardation upon radiation were determined by measuring tumor size every day (Figure 7). Co-implantation of MPR31.4 prostate cancer cells with NIH-3T3 as well as co-implantation of Py8119 prostate cancer cells with L929 led to increased tumor growth and significantly reduced tumor growth delay after IR when compared to tumors generated by cancer cells alone (Figures 7A,B,G,H). Tumors generated by co-implantation of MPR31.4 with NIH-3T3 cells showed a growth delay of only 1.57 days after receiving IR compared to the tumor generated with MPR31.4 alone which had a growth delay of 3.65 days after radiation. Moreover, the combination of Py8119 cancer cells and L929 fibroblasts led to a reduction in radiation-induced tumor growth delay of 2.94 days compared to a radiation-induced growth delay of tumors generated from Py8119 cells alone by 6.03 days (Figure 7H). However, co-implantation of MPR31.4 cells together with L929 cells as well as co-implantation of Py8119 prostate cancer cells with NIH-3T3 stromal fibroblasts had no impact on the tumor radiation response as compared to respective tumor cells implanted alone (Figures 7C–F).

**Figure 8:**
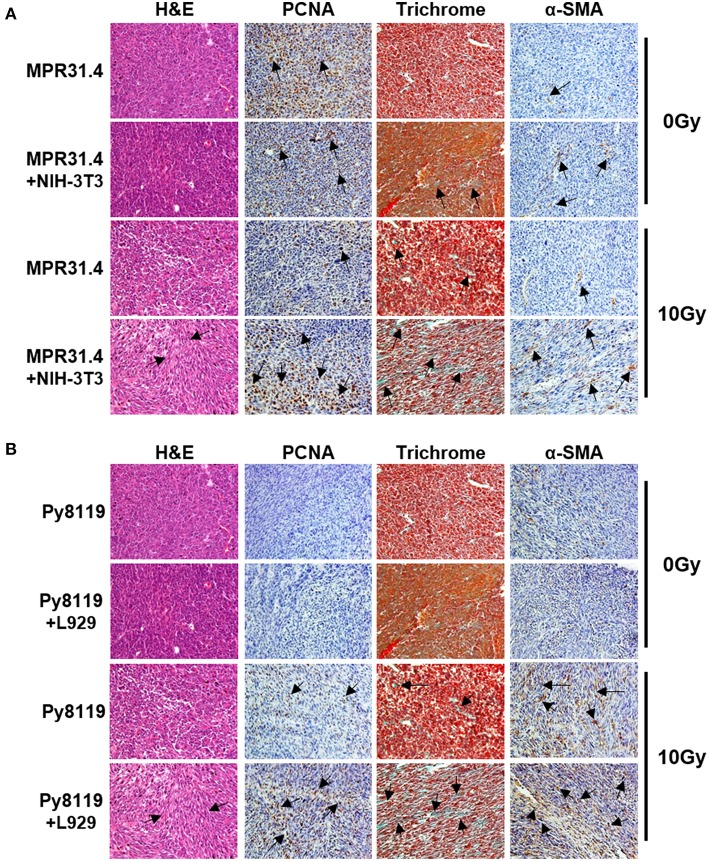
Stromal fibroblasts led to a strong increase of proliferation, fibrotic compartment as well as CAFs marker, α-SMA in the tumor after irradiation. MPR31.4 prostate cancer cells alone or together with NIH-3T3 fibroblasts [ratio of (1+1)] **(A)** and Py8119 breast cancer cells alone or together with L929 fibroblasts [ratio of (1+1)] **(B)** were subcutaneously co-implanted onto C57BL/6 mice. When tumor volumes reached a critical size (5–12 days after tumor irradiation) tumors were isolated and subjected for PCNA, α-SMA IHC, Masson Goldner trichrome and HE stains. Representatives' pictures were shown from 2 to 3 experiments (5 mice total).

Isolated tumors were further subjected for immunohistochemistry (Figure 8). Irradiated MPR31.4 tumors co-implanted with NIH-3T3 fibroblasts (Figure 8A) and Py8119 tumors co-implanted with L929 fibroblasts (Figure 8B) were more reactive for the proliferating cell nuclear antigen (PCNA) than respective tumors generated by MPR31.4 and Py8119 alone. This indicates that these fibroblasts might promote MPR31.4 or Py8119 tumor cell proliferation after radiation *in vivo*. In addition, tissue morphology and the composition of the tumors were evaluated by trichrome (TC) and Hematoxylin and Eosin (H&E) staining. Tumors generated after co-implantation of MPR31.4 cells with NIH-3T3 fibroblasts and Py8119 cells with L929 fibroblasts showed a pronounced increased in the connective tissue compartment, as visualized by the increased green-stained connective tissue areas compared to the tumors generated with cancer cells alone (Figures 8A,B). Furthermore, expression levels of the major CAF marker α-SMA showed highly increased immunoreactivity in fibroblast/CAF raised tumors after radiation as compared to the irradiated tumors raised from respective tumor cells alone (Figures 8A,B).

These results suggest that activated pro-tumorigenic CAFs might contribute to increased radioresistance of tumors. The more radioresistant MPR31.4 prostate tumors raised from co-implantation with NIH-3T3 fibroblasts as well as the Py8119 tumors raised from co-implantation with L929 fibroblasts contained a more reactive fibroblastic stroma and presumably more activated fibroblasts, in particular radioresistance-promoting CAFs after radiation whereas MPR31.4 and Py8119 might contain less activated stroma.

## Discussion

**Figure 9:**
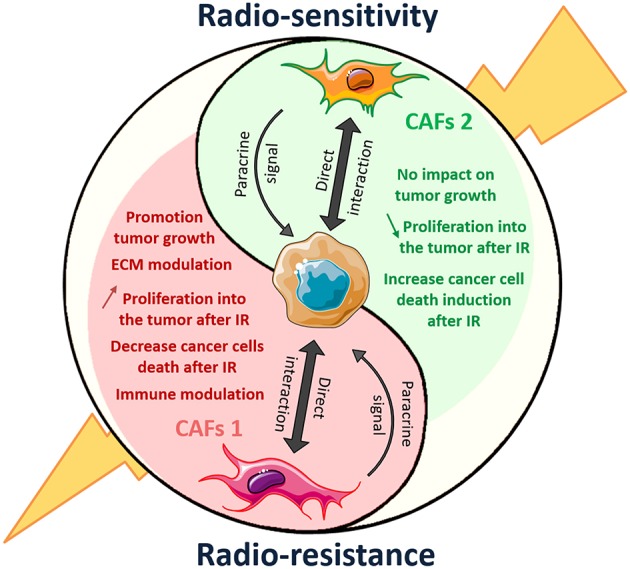
Dual role of CAFs on radiation response of solid tumor: another polarized cell type of the tumor microenvironment. CAFs 1 and 2 mark the end of the polarization spectrum represents distinct cellular lineage associated with different markers and opposing activities in the tumor. CAFs1 (in red) showed promotion of tumor growth and radiation resistance. When CAFs2 another type of CAFs, induced tumor-suppressive effect.

So far, the relevance of fibroblasts and CAFs for tumor progression and/or therapy resistance remained controversial. Here we used an unbiased approach to gain more insight if and how similar fibroblasts alter tumor progression as well as the radiation response of cells from different tumor entities. We identified two fibroblast-tumor cell combinations where fibroblasts promoted the long-term survival of the irradiated cancer cells in 2D and 3D co-culture *in vitro* as well as *in vivo*. The indirect and direct interaction of NIH-3T3 fibroblasts with MPR31.4 prostate cancer cells as well as of L929 fibroblasts with Py8119 breast cancer cells resulted an increased resistance of the cancer cells to radiation treatment. On the contrary, L929 fibroblasts in combination with MPR31.4 cells and NIH-3T3 fibroblasts with Py8119 cancer cells did not exert either a pro-tumorigenic or radioresistance-promoting effect. These findings demonstrate that the same fibroblast exerted either a tumor- and radioresistance-promoting effect, a tumor-suppressing effect or no effect (Figure 9) and point to complex bi-directional direct and indirect interactions between cancer cells and fibroblasts with impact on tumor growth and therapy outcome. We thus conclude that the impact of fibroblasts on tumor cells radiation response largely depends on the fibroblast and tumor cell type, the culture conditions (direct/indirect co-culture) and the respective endpoint (short-term vs. long-term; *in vitro* vs. *in vivo*).

Fibroblasts have been used as feeder cells in radiation biology to support the growth of target cells, particularly at low or clonal densities, by releasing growth factors to condition the medium (Puck and Marcus, 1955; Llames et al., 2015). This of course demonstrates the fibroblasts' growth and survival promoting effects. However, our findings go beyond this observation and support the current assumption that presence of adjacent tumor cells modulates fibroblast function (Madar et al., 2013). Interestingly positive and negative effects of specific fibroblasts on proliferation, and survival of certain non-irradiated cancer cells in indirect co-culture *in vitro* as well as on cancer cell survival upon irradiation could be observed. Instead only radiation-resistance promoting effects were observed in long-term survival assays, which might be related to the cancer cells potential to activate and or transform fibroblasts toward CAFs. At the early stage, fibroblasts can prevent cancer progression by specialized intercellular connections (GAP junction) between themselves (Martin et al., 1991; Klein, 2014). Fibroblast-fibroblast connections can physically prevent cancer progression by three-dimensional networking and thus affecting ECM stability as well as by paracrine signaling, e.g., secretion of tumor necrosis factor α (TNFα) and IL-6 (Alkasalias et al., 2018). At later stages however, cancer cells reprogram and activate these cells toward CAFs and thereby hijack the normal fibroblast function in support of their growth e.g., by regulating the expression of α-SMA which is an important constituent of CAFs (Desmoulière et al., 1993; Kojima et al., 2010). CAFs in turn were shown to induce cancer progression as well as resistance to cancer therapies through secretion of proteins, exosomes, and ECM remodeling factors (Son et al., 2017). Finally the interactions between fibroblast/ CAFs and cancer cells were characterized as bi-directional that promote tumor growth, cancer invasion, metastasis, and therapy resistance (De Wever et al., 2014; Alexander and Cukierman, 2016).

Thus, the tumor progression and therapy resistance-modulating activity of fibroblasts may depend on the potential of tumor cells and/or recruited immune cells to activate fibroblasts toward a CAF phenotype (Kuzet and Gaggioli, 2016). In addition, damage from radiation treatment leads to effects on numerous cell types within the tumor stroma. Precisely, radiation can lead to a quick, total and persistent activation of the TME (Barcellos-Hoff, 1998). Many cytokines are induced by IR (e.g., epidermal growth factor Dent et al., 2003 and pro-inflammatory cytokines McBride et al., 2004) and it has been shown that exposure of fibroblasts to growth factors (TGF-β, PDGF), cytokines (e.g., IL-1, IL-6) and ROS or a rigid matrix can induce a CAF phenotype (Giannoni et al., 2011; Calvo et al., 2013). CAF activation (illustrated by a strong α-SMA expression in the tumor) following radiation leads to secretion of altered growth factors and to the release of numerous ECM modulators (Barcellos-Hoff et al., 2005). Example irradiated lung and pancreatic CAFs have been reported to show changes in secretory signal with consequence for tumor growth and invasion (Milas et al., 1988; Hellevik et al., 2013). Hellevick et al. showed that CAFs isolated from lung tumors and exposed to IR result in downregulation of angiogenic molecules and secretions such as stromal cell-derived factor-1 (SDF-1), angiopoietin, and thrombospondin-2 and in up- regulation of basic fibroblast growth factor (FGF) release. It was also shown that breast cancer cell grown in association with chronic irradiated fibroblasts in a three dimensional co-culture increased the malignant behavior and disease progression (Rudolph-Owen et al., 1998). The high abundance of CAFs, leukocytes and endothelial cells in rectal tumor was shown to predict RT resistance (Isella et al., 2015).

However, the reported pronounced plasticity of fibroblasts as well as the existence of various known and not unique expressed CAF markers make it hard to define and detect a “specific” phenotype. CAFs share several markers with other stromal cells, such as epithelial cells, endothelial cells, muscle cells, and mesenchymal stem cells (Kalluri and Zeisberg, 2006; Sugimoto et al., 2006). Thus, the identification and characterization of a CAF phenotype remains challenging and we can only report which markers are expressed in a given suggested CAF in a specific experimental system. Due to the lack of reliable and specific molecular fibroblast markers, detection of CAFs within the tumor requires combination of several markers. CAFs produce mesenchyme-specific proteins, illustrating their activation state, such as fibroblast activated protein (FAP), FSP-1, (also known as S100A4), vimentin and α-SMA, all typical markers for myofibroblasts (Sugimoto et al., 2006). CAF also express receptors such as PDGFRβ that are involved in autocrine signaling loops. CAFs generate in addition a variety of matrix-components and matrix-remodeling enzymes such as the chondroitin sulfate proteoglycan NG2, tenascin C (TN-C), and fibronectin (Augsten, 2014). Moreover, those markers are differently expressed from one CAFs to another (e.g., from different tissue) which indicates the existence of several sub-populations of CAFs, which are able to exert distinct tumorigenic effects (Sugimoto et al., 2006). Sugimoto et al. described different CAFs-subtypes based on the expression analysis of FSP-1, PDGRβ, NG2 and α-SMA in pancreatic and breast cancer mouse models (Sugimoto et al., 2006). This study revealed that one CAFs-subtype expressed α-SMA, PDGRβ, and NG2, while another expressed FSP-1. However, it is not clear how many CAF subtypes in a certain cancer entity exist.

In the present study, it was decided to characterize the different CAFs depending of their action on the tumor radiation response. NIH-3T3 fibroblasts that induced MPR31.4 prostate tumor radioresistance and L929 fibroblasts which induced Py8119 breast tumors radioresistance were expressing different CAF markers profiles than L929 fibroblasts, when co-cultured with MPR31.4 prostate cancer cells (inhibitory action) or NIH-3T3 fibroblasts which had no impact on Py8119 breast cancer cells and tumors. However, a clear and specific CAF phenotype was not induced in the different fibroblasts *in vitro*. However, tumors raised from MPR31.4 tumor cells with NIH-3T3 fibroblasts and tumors from Py8119 tumor cells with L929 fibroblasts, contained more reactive stroma and activated pro-tumorigenic CAFs after radiation than tumors generated from MPR31.4 and Py8119 tumor cells alone. These activated pro-tumorigenic CAFs might contribute to increased radio-resistance of the tumors. Thus, the hitherto established CAFs markers showed very variable expression levels in the different CAF entities, which strongly indicates the needs for the identification of additional phenotype markers between the different fibroblasts and respective CAFs. In line with our observations, CAFs are generally defined by their association with cancer cells within a tumor, because the well-known candidate markers for CAFs differ in their relative expression, abundance, and distinct overlapping expression patterns in different tissue types because the tumor cells seem to activate the fibroblasts differentially (Kalluri, 2016; LeBleu and Kalluri, 2018). In addition, the difference between a CAF and a normal fibroblast in the tumor microenvironment can be considered as functional, rather than defined by the specific expression of a certain biological marker (Nurmik et al., 2019).

CAF heterogeneity as revealed by their variable phenotype and differential functionality might be also related to their origin. Resident fibroblasts were often admitted as the main source of CAFs, as their low proliferative capacity challenged the model of local fibroblast activation as unique CAF source (Kojima et al., 2010). However, the precise origin of CAFs remains elusive. CAFs were shown to be derived from different precursor cells: In addition to the expansion of resident, hitherto quiescent fibroblasts when cancer arises (LeBleu and Kalluri, 2018), CAFs were shown to be recruited from other sources, e.g., the bone marrow, as bone marrow-derived mesenchymal and hematopoietic stem cells could differentiate into CAFs (Hinz et al., 2007; LeBleu et al., 2013; Madar et al., 2013). Fibrocytes from the bone marrow can also be recruited and differentiate into myofibroblasts (Gomperts and Strieter, 2007; Moore and Kolb, 2014). Additionally, CAFs might derive from other stromal cells as a result of trans-differentiation of pericytes, endothelial (Zeisberg et al., 2007), or adipocytes (Xiong et al., 2015). Likewise, epithelial cells can adopt a fibroblasts-like phenotype by a epithelial-mesenchymal transition (EMT) (Iwano et al., 2002). However, due to the absence of unique fibroblast/CAF markers, the precise identification of their biological origin remains a major challenge (Ziani et al., 2018).

In general normal fibroblasts as well as CAF have stable karyotype and a lack of genetic alterations (Qiu et al., 2008; Walter et al., 2008; Hosein et al., 2010; Öhlund et al., 2014). Thus, these cells are genetically stable and do not cause cancer, but in tumors it is clear that CAFs affect tumor progression and the response to applied cancer therapy. CAFs-derived signals (e.g., MMPs) were herein shown to promote the adoption of a cancer stem cell phenotype via EMT induction, which in turn leads to more cancer aggressiveness (Giannoni et al., 2010; Fiaschi et al., 2013), and thus might be one tumor radio-resistance mechanism taking place. However, in our indirect co-culture system, only L929 fibroblasts induced an EMT phenotype in MPR31.4 and Py8119 cancer cells after radiation. Whereas, NIH-3T3 fibroblasts showed to induce radiation resistance of MPR31.4, it did not induce EMT in MPR31.4 after radiation. In addition, L929 fibroblasts had no influence on the radiation response of MPR31.4, however it induced EMT in MPR31.4's (Supplementary Figure 2). Therefore, the differential impact of fibroblasts on the cancer cell radiation response could not be related to cancer cells EMT induction.

Pro-tumorigenic CAFs however can secrete a variety of pro-inflammatory factors (Erez et al., 2010, 2013) leading to the recruitment and promotion of immunosuppressive (Mace et al., 2013) and tumor promoting immune cells (Comito et al., 2014) which then contribute to a tumor permissive environment. Tumor progression requires the cancer cells to develop resistance to immune attack. Thus, immune cells might play a role in the CAFs-induced tumor radio-resistance phenotype. A first look at the potential role of immune cell infiltration using immunohistochemistry and CD45-immunoreactivity of tumor sections generated by co-implantation of the radioresistant MPR31.4 cells with NIH-3T3 fibroblasts and Py8119 cells with L929 fibroblasts combinations was performed. After IR there were more CD45+ cells detectable in tissue-specimen as compared to respective unirradiated tumors and tumors generated from cancer cells alone (Supplementary Figure 3). Thus, there is already an indication that immune infiltration induced by radiation and CAFs may play a role in the tumor radiation response. Comito et al. could already show that CAFs support the differentiation of macrophages into tumor-promoting TAM-2 in prostate tumors (Comito et al., 2014). Here CAFs were shown to contribute to the recruitment of immunosuppressive and tumor promoting immune cells in the prostate and breast tumors after radiation, which in turn could contribute to the protection of the tumor from radiation. However, a detailed analysis of the CAFs potential to affect and recruit immune cells as well as the analyses of the nature of infiltrated immune cells needs to be performed in future studies.

Conclusively, the tumor microenvironment and in particularly the CAFs play a dominant role in the radiation response of solid tumors, where the cancer cells (“seed”) form their own microenvironment (“soil”). As a result, more work has to be done to discriminate the normal fibroblasts from CAFs and to unravel the phenotype and functionality of CAFs in a tumor entity-specific manner to successfully target these cells or their resistance-promoting traits for improving the outcome of cancer therapy.

## Data Availability

All datasets generated for this study are included in the manuscript/Supplementary Files.

## Ethics Statement

The animal study was reviewed and approved by Landesamt für Natur, Umwelt und Verbraucherschutz (LANUV), Regierungspräsidium Düsseldorf.

## Author Contributions

AS and DK performed experiments. AS analyzed results and made the figures. NC provided materials. DK and VJ designed research. VJ performed fund raising. AS, VJ, and DK wrote the paper. All authors reviewed and approved the manuscript.

### Conflict of Interest Statement

The authors declare that the research was conducted in the absence of any commercial or financial relationships that could be construed as a potential conflict of interest.

## References

[B1] Alexander J. Cukierman E. (2016). Stromal dynamic reciprocity in cancer: intricacies of fibroblastic-ECM interactions. Curr. Opin. Cell Biol. 42, 80–93. 10.1016/j.ceb.2016.05.002 27214794 PMC5064819

[B2] Alkasalias T. Moyano-Galceran L. Arsenian-Henriksson M. Lehti K. Alkasalias T. Moyano-Galceran L. (2018). Fibroblasts in the tumor microenvironment: shield or spear? Int. J. Mol. Sci. 19:1532 10.3390/ijms19051532 PMC5983719 29883428

[B3] Augsten M. (2014). Cancer-associated fibroblasts as another polarized cell type of the tumor microenvironment. Front. Oncol. 4:62. 10.3389/fonc.2014.00062 24734219 PMC3973916

[B4] Barcellos-Hoff M. H. (1998). The potential influence of radiation-induced microenvironments in neoplastic progression. J. Mammary Gland Biol. Neoplasia 3, 165–175. 10.1023/A:1018794806635 10819525

[B5] Barcellos-Hoff M. H. Park C. Wright E. G. (2005). Radiation and the microenvironment – tumorigenesis and therapy. Nat. Rev. Cancer 5, 867–875. 10.1038/nrc1735 16327765

[B6] Bissell M. J. Radisky D. (2001). Putting tumours in context. Nat Rev Cancer 1, 46–54. 10.1038/35094059 11900251 PMC2975572

[B7] Bonomi A. Sordi V. Dugnani E. Ceserani V. Dossena M. Coccè V.. (2015). Gemcitabine-releasing mesenchymal stromal cells inhibit *in vitro* proliferation of human pancreatic carcinoma cells. Cytotherapy, 17, 1687–1695. 10.1016/j.jcyt.2015.09.005 26481416

[B8] Calvo F. Ege N. Grande-Garcia A. Hooper S. Jenkins R. P. Chaudhry S. I.. (2013). Mechanotransduction and YAP-dependent matrix remodelling is required for the generation and maintenance of cancer-associated fibroblasts. Nat. Cell Biol. 15, 637–646. 10.1038/ncb2756 23708000 PMC3836234

[B9] Comito G. Giannoni E. Segura C. P. Barcellos-de-Souza P. Raspollini M. R. Baroni G.. (2014). Cancer-associated fibroblasts and M2-polarized macrophages synergize during prostate carcinoma progression. Oncogene 33, 2423–2431. 10.1038/onc.2013.191 23728338

[B10] Dauer P. Zhao X. Gupta V. K. Sharma N. Kesh K. Gnamlin P.. (2018). Inactivation of cancer-associated-fibroblasts disrupts oncogenic signaling in pancreatic cancer cells and promotes its regression. Cancer Res. 78, 1321–1333. 10.1158/0008-5472.CAN-17-2320 29259015 PMC5935584

[B11] De Wever O. Van Bockstal M. Mareel M. Hendrix A. Bracke M. (2014). Carcinoma-associated fibroblasts provide operational flexibility in metastasis. Semin. Cancer Biol. 25, 33–46. 10.1016/j.semcancer.2013.12.009 24406210

[B12] Demaria S. Bhardwaj N. McBride W. H. Formenti S. C. (2005). Combining radiotherapy and immunotherapy: a revived partnership. Int. J. Radi. Oncol. Biol. Phys. 63, 655–666. 10.1016/j.ijrobp.2005.06.032 16199306 PMC1489884

[B13] Dent P. Yacoub A. Fisher P. B. Hagan M. P. Grant S. (2003). MAPK pathways in radiation responses. Oncogene 22, 5885–5896. 10.1038/sj.onc.1206701 12947395

[B14] Desmoulière A. Geinoz A. Gabbiani F. Gabbiani G. (1993). Transforming growth factor-beta 1 induces alpha-smooth muscle actin expression in granulation tissue myofibroblasts and in quiescent and growing cultured fibroblasts. J. Cell Biol. 122, 103–111. 10.1083/jcb.122.1.103 8314838 PMC2119614

[B15] Dudás J. Fullár A. Bitsche M. Schartinger V. Kovalszky I. Sprinzl G. M. (2011). Tumor-produced, active interleukin-1 β regulates gene expression in carcinoma-associated fibroblasts. Exp. Cell Res. 317, 2222–2229. 10.1016/j.yexcr.2011.05.023 21664353 PMC3171161

[B16] Duluc C. Moatassim-Billah S. Chalabi-Dchar M. Perraud A. Samain R. Breibach F.. (2015). Pharmacological targeting of the protein synthesis mTOR/4E-BP1 pathway in cancer-associated fibroblasts abrogates pancreatic tumour chemoresistance. EMBO Mol. Med. 7, 735–753. 10.15252/emmm.201404346 25834145 PMC4459815

[B17] Durand R. E. (1994). The influence of microenvironmental factors during cancer therapy. In Vivo 8, 691–702. 7727714

[B18] Eke I. Zscheppang K. Dickreuter E. Hickmann L. Mazzeo E. Unger K.. (2015). Simultaneous beta1 integrin-EGFR targeting and radiosensitization of human head and neck cancer. J. Natl. Cancer Inst. 107:2. 10.1093/jnci/dju419 25663685

[B19] Erez N. Glanz S. Raz Y. Avivi C. Barshack I. (2013). Cancer associated fibroblasts express pro-inflammatory factors in human breast and ovarian tumors. Biochem. Biophys. Res. Commun. 437, 397–402. 10.1016/j.bbrc.2013.06.089 23831470

[B20] Erez N. Truitt M. Olson P. Hanahan D. (2010). Cancer-associated fibroblasts are activated in incipient neoplasia to orchestrate tumor-promoting inflammation in an NF-κB-dependent manner. Cancer Cell 17, 135–147. 10.1016/j.ccr.2009.12.041 20138012

[B21] Fiaschi T. Giannoni E. Taddei M. L. Cirri P. Marini A. Pintus G.. (2013). Carbonic anhydrase IX from cancer-associated fibroblasts drives epithelial-mesenchymal transition in prostate carcinoma cells. Cell Cycle 12, 1791–1801. 10.4161/cc.24902 23656776 PMC3713137

[B22] Franco O. E. Shaw A. K. Strand D. W. Hayward S. W. (2010). Cancer associated fibroblasts in cancer pathogenesis. Semin. Cell Dev. Biol. 21, 33–39. 10.1016/j.semcdb.2009.10.010 19896548 PMC2823834

[B23] Furuta S. Ghajar C. M. Bissell M. J. (2011). Caveolin-1: would-be achilles' heel of tumor microenvironment? Cell Cycle 10:3431. 10.4161/cc.10.20.17648 22030625

[B24] Giannoni E. Bianchini F. Calorini L. Chiarugi P. (2011). Cancer associated fibroblasts exploit reactive oxygen species through a proinflammatory signature leading to epithelial mesenchymal transition and stemness. Antioxid. Redox Signal. 14, 2361–2371. 10.1089/ars.2010.3727 21235356

[B25] Giannoni E. Bianchini F. Masieri L. Serni S. Torre E. Calorini L.. (2010). Reciprocal activation of prostate cancer cells and cancer-associated fibroblasts stimulates epithelial-mesenchymal transition and cancer stemness. Cancer Res. 70, 6945–6956. 10.1158/0008-5472.CAN-10-0785 20699369

[B26] Gomperts B. N. Strieter R. M. (2007). Fibrocytes in lung disease. J. Leukoc. Biol. 82, 449–456. 10.1189/jlb.0906587 17550974

[B27] Hanahan D. Robert Weinberg A. (2011). Hallmarks of cancer, the next generation. Cell 144, 646–674. 10.1016/j.cell.2011.02.013 21376230

[B28] Hanahan D. Weinberg R. A. (2000). The hallmarks of cancer. Cell 100, 57–70. 10.1016/S0092-8674(00)81683-9 10647931

[B29] Hawinkels L. J. A. C. Paauwe M. Verspaget H. W. Wiercinska E. J. M. van der Zon K. van der Ploeg K.. (2014). Interaction with colon cancer cells hyperactivates TGF-β signaling in cancer-associated fibroblasts. Oncogene 33, 97–107. 10.1038/onc.2012.536 23208491

[B30] Hellevik T. Pettersen I. Berg V. Bruun J. Bartnes K. Busund L.-T. Martinez-Zubiaurre, I.. (2013). Changes in the secretory profile of NSCLC-associated fibroblasts after ablative radiotherapy: potential impact on angiogenesis and tumor growth. Transl. Oncol. 6, 66–74. 10.1593/tlo.12349 23418618 PMC3573655

[B31] Hesler R. A. Huang J. J. Starr M. D. Treboschi V. M. Bernanke A. G. Nixon A. B.. (2016). TGF-β-induced stromal CYR61 promotes resistance to gemcitabine in pancreatic ductal adenocarcinoma through downregulation of the nucleoside transporters hENT1 and hCNT3. Carcinogenesis 37, 1041–1051. 10.1093/carcin/bgw093 27604902 PMC5091039

[B32] Hinz B. Phan S. H. Thannickal V. J. Galli A. Bochaton-Piallat M. L. Gabbiani G. (2007). The myofibroblast: One function, multiple origins. Am. J. Pathol. 170, 1807–1816. 10.2353/ajpath.2007.070112 17525249 PMC1899462

[B33] Hosein A. N. Wu M. Arcand S. L. Lavallée S. Hébert J. Tonin P. N. (2010). Breast carcinoma-associated fibroblasts rarely contain p53 mutations or chromosomal aberrations. Cancer Res. 70, 5770–5777. 10.1158/0008-5472.CAN-10-0673 20570891

[B34] Isella C. Terrasi A. Bellomo S. E. Petti C. Galatola G. Muratore A.. (2015). Stromal contribution to the colorectal cancer transcriptome. Nat. Genet. 47, 312–319. 10.1038/ng.3224 25706627

[B35] Ito E. Yue S. Moriyama E. H. Hui A. B. Kim I. Shi W.. (2011). Uroporphyrinogen decarboxylase is a radiosensitizing target for head and neck cancer. Sci. Transl. Med. 3:67ra7. 10.1126/scitranslmed.3001922 21270338

[B36] Iwano M. Plieth D. Danoff T. M. Xue C. Okada H. Neilson E. G. (2002). Evidence that fibroblasts derive from epithelium during tissue fibrosis. J. Clin. Invest. 110, 341–350. 10.1172/JCI0215518 12163453 PMC151091

[B37] Jiang G. M. Xu W. Du J. Zhang K. S. Zhang Q. G. Wang X. W.. (2016). The application of the fibroblast activation protein α-targeted immunotherapy strategy. Oncotarget 7, 33472–33482. 10.18632/oncotarget.8098 26985769 PMC5078111

[B38] Kaler P. Owusu B. Y. Augenlicht L. Klampfer L. (2014). The role of STAT1 for crosstalk between fibroblasts and colon cancer cells. Front. Oncol. 4:88. 10.3389/fonc.2014.00088 24818101 PMC4012204

[B39] Kalluri R. (2016). The biology and function of fibroblasts in cancer. Nat. Rev. Cancer 16, 582–598. 10.1038/nrc.2016.73 27550820

[B40] Kalluri R. Zeisberg M. (2006). Fibroblasts in cancer. Nat. Rev. Cancer 6, 392–401. 10.1038/nrc1877 16572188

[B41] Ketteler J. Klein D. (2018). Caveolin-1, cancer and therapy resistance. Int. J. Cancer 143, 2092–2104. 10.1002/ijc.31369 29524224

[B42] Ketteler J. Panic A. Reis H. Wittka A. Maier P. Herskind C.. (2019). Progression-related loss of stromal caveolin 1 levels mediates radiation resistance in prostate carcinoma via the apoptosis inhibitor TRIAP1. J. Clin. Med. 8:3. 10.3390/jcm8030348 30871022 PMC6462938

[B43] Klein D. Schmitz T. Verhelst V. Panic A. Schenck M. Reis H.. (2015). Endothelial Caveolin-1 regulates the radiation response of epithelial prostate tumors. Oncogenesis 4:e148. 10.1038/oncsis.2015.9 25985209 PMC4450264

[B44] Klein D. Steens J. Wiesemann A. Schulz F. Kaschani F. Rock K.. (2017). Mesenchymal stem cell therapy protects lungs from radiation-induced endothelial cell loss by restoring superoxide dismutase 1 expression. Antioxid. Redox Signal. 26, 563–582. 10.1089/ars.2016.6748 27572073 PMC5393411

[B45] Klein G. (2014). Evolutionary aspects of cancer resistance. Semin. Cancer Biol. 25, 10–14. 10.1016/j.semcancer.2014.01.001 24440448

[B46] Kojima Y. Acar A. Eaton E. N. Mellody K. T. Scheel C. Ben-Porath I.. (2010). Autocrine TGF-beta and stromal cell-derived factor-1 (SDF-1) signaling drives the evolution of tumor-promoting mammary stromal myofibroblasts. Proc. Natl. Acad. Sci. U.S.A. 107, 20009–20014. 10.1073/pnas.1013805107 21041659 PMC2993333

[B47] Kuzet S. E. Gaggioli C. (2016). Fibroblast activation in cancer: when seed fertilizes soil. Cell Tissue Res. 365, 607–619. 10.1007/s00441-016-2467-x 27474009

[B48] LeBleu V. S. Kalluri R. (2018). A peek into cancer-associated fibroblasts: origins, functions and translational impact. Dis. Model Mech. 11:dmm029447. 10.1242/dmm.029447 29686035 PMC5963854

[B49] LeBleu V. S. Taduri G. O'Connell J. Teng Y. Cooke V. G. Woda C.. (2013). Origin and function of myofibroblasts in kidney fibrosis. Nat. Med. 19, 1047–1053. 10.1038/nm.3218 23817022 PMC4067127

[B50] Leef G. Thomas S. M. (2013). Molecular communication between tumor-associated fibroblasts and head and neck squamous cell carcinoma. Oral Oncol. 49, 381–386. 10.1016/j.oraloncology.2012.12.014 23357526 PMC3615086

[B51] Lewis M. P. Lygoe K. A. Nystrom M. L. Anderson W. P. Speight P. M. Marshall J. F.. (2004). Tumour-derived TGF-β1 modulates myofibroblast differentiation and promotes HGF/SF-dependent invasion of squamous carcinoma cells. Br. J. Cancer 90, 822–832. 10.1038/sj.bjc.6601611 14970860 PMC2410183

[B52] Llames S. Garcia-Perez E. Meana A. Larcher F. del Rio M. (2015). Feeder layer cell actions and applications. Tissue Eng. B Rev. 21, 345–353. 10.1089/ten.teb.2014.0547 25659081 PMC4533020

[B53] Mace T. A. Ameen Z. Collins A. Wojcik S. Mair M. Young G. S.. (2013). Pancreatic cancer-associated stellate cells promote differentiation of myeloid-derived suppressor cells in a STAT3-dependent manner. Cancer Res. 73, 3007–3018. 10.1158/0008-5472.CAN-12-4601 23514705 PMC3785672

[B54] Madar S. Goldstein I. Rotter V. (2013). ‘Cancer associated fibroblasts’ – more than meets the eye. Trends Mol. Med. 19, 447–453 10.1016/j.molmed.2013.05.004 23769623

[B55] Martin W. Zempel G. Hülser D. Willecke K. (1991). Growth inhibition of oncogene-transformed rat fibroblasts by cocultured normal cells: relevance of metabolic cooperation mediated by gap junctions. Cancer Res. 51, 5348–5351. 1913656

[B56] McBride W. H. Chiang C. S. Olson J. L. Wang C. C. Hong J. H. Pajonk F.. (2004). A sense of danger from radiation. Radiat. Res. 162, 1–19. 10.1667/RR3196 15222781

[B57] Milas L. Hirata H. Hunter N. Peters L. J. Sato N. Nagai E.. (1988). Effect of radiation-induced injury of tumor bed stroma on metastatic spread of murine sarcomas and carcinomas. Cancer Res. 48, 2116–2120. 3349483

[B58] Moore B. B. Kolb M. (2014). Fibrocytes and progression of fibrotic lung disease. Ready for showtime? Am. J. Respir. Crit. Care Med. 190, 1338–1339. 10.1164/rccm.201411-2013ED 25496101

[B59] Neesse A. Michl P. Frese K. K. Feig C. Cook N. Jacobetz M. A.. (2011). Stromal biology and therapy in pancreatic cancer. Gut 60, 861–868. 10.1136/gut.2010.226092 20966025

[B60] Nurmik M. Ullmann P. Rodriguez F. Haan S. Letellier E. (2019). In search of definitions: cancer-associated fibroblasts and their markers. Int. J. Cancer. 1–11. 10.1002/ijc.32193. [Epub ahead of print]. 30734283 PMC6972582

[B61] Öhlund D. Elyada E. Tuveson D. (2014). Fibroblast heterogeneity in the cancer wound. J. Exp. Med. 211, 1503–1523. 10.1084/jem.20140692 25071162 PMC4113948

[B62] Ozdemir B. C. Pentcheva-Hoang T. Carstens J. L. Zheng X. Wu C. C. Simpson T. R. Kalluri R.. (2014). Depletion of carcinoma-associated fibroblasts and fibrosis induces immunosuppression and accelerates pancreas cancer with reduced survival. Cancer Cell 25, 719–734 10.1016/j.ccr.2014.04.005 24856586 PMC4180632

[B63] Panic A. Ketteler J. Reis H. Sak A. Herskind C. Maier P.. (2017). Progression-related loss of stromal Caveolin 1 levels fosters the growth of human PC3 xenografts and mediates radiation resistance. Sci. Rep. 7:41138. 10.1038/srep41138 28112237 PMC5255553

[B64] Pietras K. Östman A. (2010). Hallmarks of cancer: Interactions with the tumor stroma. Exp. Cell Res. 316, 1324–1331. 10.1016/j.yexcr.2010.02.045 20211171

[B65] Puck T. T. Marcus P. I. (1955). A rapid method for viable cell titration and clone production with hela cells in tissue culture: the use of X-irradiated cells to supply conditioning factors. Proc. Natl. Acad. Sci. U.S.A. 41, 432–437. 10.1073/pnas.41.7.432 16589695 PMC528114

[B66] Qiu W. Hu M. Sridhar A. Opeskin K. Fox S. Shipitsin M.. (2008). No evidence of clonal somatic genetic alterations in cancer-associated fibroblasts from human breast and ovarian carcinomas. Nat. Genet., 40, 650–655. 10.1038/ng.117 18408720 PMC3745022

[B67] Rudolph-Owen L. A. Chan R. Muller W. J. Matrisian L. M. (1998). The matrix metalloproteinase matrilysin influences early-stage mammary tumorigenesis. Cancer Res. 58, 5500–5506. 9850086

[B68] Scott A. M. Wiseman G. Welt S. Adjei A. Lee F. T. Old L. J. (2003). A Phase I dose-escalation study of sibrotuzumab in patients with advanced or metastatic fibroblast activation protein-positive cancer. Clin. Cancer Res. 9, 1639–1647. Available online at: http://clincancerres.aacrjournals.org/content/9/5/1639 12738716

[B69] Shaker M. R. Yang G. Timme T. L. Park S. H. Kadmon D. Ren C.. (2000). Dietary 4-HPR suppresses the development of bone metastasis *in vivo* in a mouse model of prostate cancer progression. Clin. Exp. Metastasis 18, 429–438. 10.1023/A:1010905309570 11467776

[B70] Smith N. R. Baker D. Farren M. Pommier A. Swann R. Wang X.. (2013). Tumor stromal architecture can define the intrinsic tumor response to VEGF-targeted therapy. Clin Cancer Res. 19, 6943–6956. 10.1158/1078-0432.CCR-13-1637 24030704

[B71] Son B. Lee S. Youn H. Kim E. Kim W. Youn B. (2017). The role of tumor microenvironment in therapeutic resistance. Oncotarget 8, 3933–3945. 10.18632/oncotarget.13907 27965469 PMC5354804

[B72] Sugimoto H. Mundel T. M. Kieran M. W. Kalluri R. (2006). Identification of fibroblast heterogeneity in the tumor microenvironment. Cancer Biol. Ther. 5, 1640–1646. 10.4161/cbt.5.12.3354 17106243

[B73] Thompson T. C. Timme T. L. Kadmon D. Park S. H. Egawa S. Yoshida K. (1993). Genetic predisposition and mesenchymal-epithelial interactions in ras + myc—induced carcinogenesis in reconstituted mouse prostate. Mol. Carcinog. 7, 165–179. 10.1002/mc.2940070307 8489712

[B74] Walter K. Omura N. Hong S. M. Griffith M. Goggins M. (2008). Pancreatic cancer associated fibroblasts display normal allelotypes. Cancer Biol. Ther. 7, 882–888. 10.4161/cbt.7.6.5869 18344687 PMC2692624

[B75] Xiong Y. McDonald L. T. Russell D. L. Kelly R. R. Wilson K. R. Mehrotra M.. (2015). Hematopoietic stem cell-derived adipocytes and fibroblasts in the tumor microenvironment. World J. Stem Cells 7, 253–265 10.4252/wjsc.v7.i2.253 25815113 PMC4369485

[B76] Zeisberg E. M. Potenta S. Xie L. Zeisberg M. Kalluri R. (2007). Discovery of endothelial to mesenchymal transition as a source for carcinoma-associated fibroblasts. Cancer Res. 67, 10123–10128. 10.1158/0008-5472.CAN-07-3127 17974953

[B77] Zhang H. Wu H. Guan J. Wang L. Ren X. Shi X.. (2015). Paracrine SDF-1alpha signaling mediates the effects of PSCs on GEM chemoresistance through an IL-6 autocrine loop in pancreatic cancer cells. Oncotarget 6, 3085–3097. 10.18632/oncotarget.3099 25609203 PMC4413639

[B78] Ziani L. Chouaib S. Thiery J. (2018). Alteration of the antitumor immune response by cancer-associated fibroblasts. Front. Immunol. 9:414. 10.3389/fimmu.2018.00414 29545811 PMC5837994

