# Supplementary material for: Impact of Cancer-Associated Fibroblast on the Radiation-Response of Solid Xenograft Tumors
Source: Front Mol Biosci. 2019 Aug 13;6:70. doi: 10.3389/fmolb.2019.00070 (PMC6705217; doi:10.3389/fmolb.2019.00070)
Supplement: Supplementary file 1 [file Data_Sheet_1.PDF]

Supplementary Figures

Supplementary Figure 1

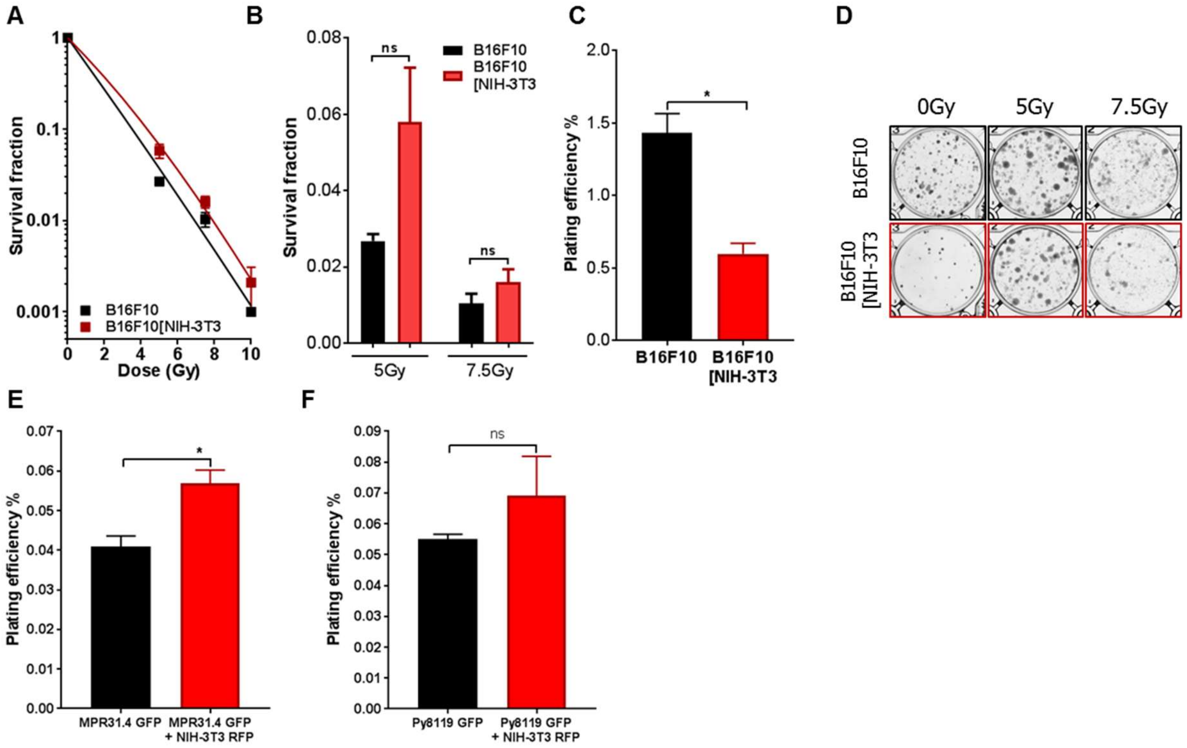

Supplementary Figure 1: **NIH-3T3 fibroblasts did not alter the clonogenic survival of B16F10 melanoma cancer cells but induced a decrease in B16F10 colonies formation in indirect co-culture**. B16F10 cancer cells were plated alone or together with stromal NIH-3T3 fibroblasts for 24h prior to irradiation with 0 or 10Gy (ratio 1+1) and further incubated for additional 7 days after irradiation. Graphs depict the surviving fractions from two independent experiments measured in sextuplet each (means ± SEM) (**A, B).** Plating efficiency of the cells **(C).** Plates were scanned, colonies were counted, and survival fraction was calculated (**D**). "ns" present for no significant and *p<0.05 analyzed by one-way ANOVA test followed by Tukey's test. NIH-3T3 fibroblasts induced an increase of MPR31.4 colonies formation in 3D direct co-culture but not of Py8119. MPR31.4 GFP and Py8119 GFP cancer cells were plate alone or together with NIH-3T3 RFP fibroblasts i na 3D Matrigel system for colonies formation assay. Plating efficiency of the cells were calculated (**E-F**). "ns" present for no significant and *p<0.5 analyzed by t-test.

Supplementary Figure 2

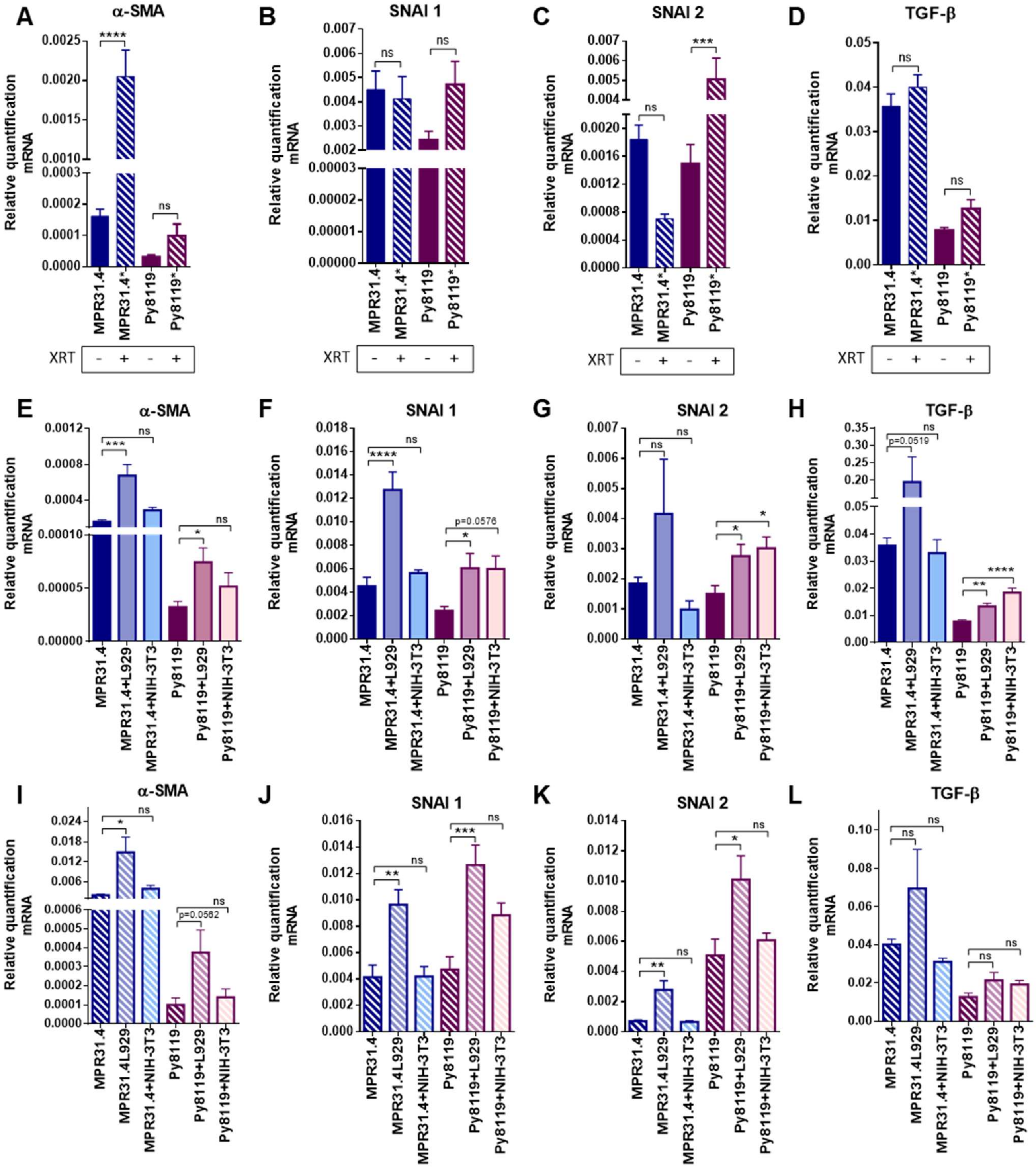

Supplementary Figure 2: **L929 fibroblasts-derived signals induced EMT in cancer cells after IR.** MPR31.4 or Py8119 cancer cells alone (**A-D**) or together with L929 or NIH-3T3 stromal fibroblasts (transwell, (ratio 1+1)) were cultured 24h prior irradiation with 0 (**E-H**) or 10Gy (**I-L**). After 72h, qRT-PCR analysis of EMT markers α-SMA (Acta2), SNAI1, SNAI2 and TGF-β were performed in total RNA isolates of cultured cancer cells. Respective expression levels were normalized to β-actin (set at 1). Shown are mean values ± SEM from 3 independent samples per group measured each in triplicate each. "ns" present for no significant, *p<0.05, **p<0.01, ***p<0.001 and ****p<0.00 analyzed by one-way ANOVA test followed by Tukey's test.

Supplementary Figure 3

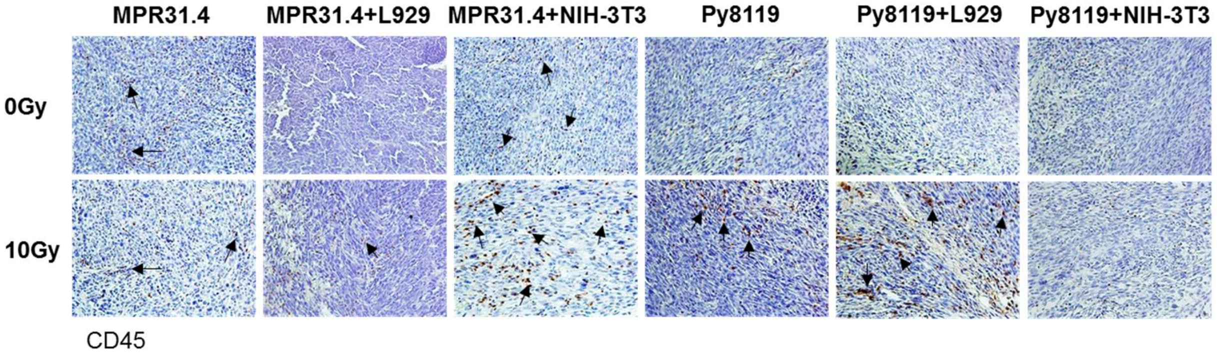

Supplementary Figure 3: **NIH-3T3 fibroblasts might account for an increase of immune cell infiltration into MPR31.4 tumors after irradiation as well as L929 fibroblasts into Py8119 tumors**. MPR31.4 prostate or Py8119 breast cancer cells alone or together with NIH-3T3 or L929 fibroblasts (ratio of (1+1)) were subcutaneously co-implanted onto C57BL/6 mice. When tumor volumes reached a critical size (5–21 days after tumor irradiation) tumors were isolated and subjected for CD45 IHC staining. Representatives' pictures were shown from 2-3 experiments (5 mice per group).
